# Development and Evaluation of a New Self-Administered Near Visual Acuity Chart: Accuracy and Feasibility of Usage

**DOI:** 10.3390/jcm13072064

**Published:** 2024-04-02

**Authors:** Hadas Ben-Eli, Eyal Banin, Jaime Levy, Miryam Glik, Sarah Afriat, Yasmin Magal, Rivka Harari, Aviya Benyamin, Shira Shein, Itay Chowers

**Affiliations:** 1 Department of Ophthalmology, Hadassah-Hebrew University Medical Center, P.O. Box 12000, Jerusalem 91120, Israelljaime@hadassah.org.il (J.L.); yasmin.magal@mail.huji.ac.il (Y.M.); rivka655@gmail.com (R.H.); shines@hadassah.org.il (S.S.); chowers@hadassah.org.il (I.C.); 2 Department of Optometry and Vision Science, Hadassah Academic College, 37 Neviim St., Jerusalem 9101001, Israel; miriammeltzer16@gmail.com (M.G.); sarahrachel.m@gmail.com (S.A.); aviyaben66@gmail.com (A.B.)

**Keywords:** visual acuity, near chart, self-assessment, development, validation

## Abstract

**Background**: Visual acuity (VA) assessments are crucial in ophthalmology but traditionally rely on in-clinic evaluations. The emergence of telemedicine has spurred interest in creating dependable self-administered VA tests for use beyond standard clinical environments. This study evaluated the practicality and validity of a self-administered near VA card test against traditional Snellen and Rosenbaum Pocket Vision Screener (RPVS) methods for home monitoring and enhancing clinical workflow. **Methods**: In a cross-sectional study, a near VA card (Hadassah Self-Visual Acuity Screener (HSVA)) was developed with written and videotaped instructions for self-use. Patients with a minimal best-corrected VA (BCVA) of 1.0 LogMAR in at least one eye were recruited from ophthalmology and optometry clinics. Outcomes included the mean BCVA difference between the self-administered values and those obtained by the examiner, and correlations between BCVA values obtained by the Snellen, RPVS, HSVA, and previous distance BCVA methods according to the patients’ electronic medical records. **Results**: A total of 275 participants (mean age: 42.5 ± 19.4 years; range: 18–89 years; 47% female) were included. Test–retest reliability analysis of the HSVA demonstrated a very good correlation and repeatability (n = 38 patients; Rs = 1.0; *p* < 0.001). Accuracy analysis revealed the mean LogMAR BCVA values of an additional 237 patients obtained by the Snellen, RPVS, and HSVA methods were similar (*p* = 0.10). The self-test BCVA results obtained by the HSVA agreed with the masked examiner-tested VA results (n = 67 patients; *p* = 0.17; Rs = 0.87; ICC = 0.96). Similar results were obtained when stratification by median age (42 years) was performed. Bland–Altman analysis of the HSVA and RPVS methods demonstrated a good agreement. To assess whether the HSVA could predict the VA results in the clinically used charts, multivariate analysis was used and revealed that the HSVA predicted the RPVS results (β = 0.91; *p* = 0.001; R^2^ = 0.88), and the self-test HSVA predicted the Snellen VA results within two lines (β = 0.93; *p* = 0.01; R^2^ = 0.36). **Conclusions**: The home-based HSVA assessment exhibited high test–retest reliability, accuracy, and alignment with clinical-standard VA tests. Its efficacy in self-testing mirrored examiner-conducted VA assessments and accurately predicted Snellen VA outcomes, indicating the HSVA’s suitability for self-monitoring in chronic ocular conditions or when access to conventional examinations is limited. The utility of self-administered VA tests may extend beyond ophthalmology and optometry, potentially benefiting primary care, emergency medicine, and neurology. Further research is needed to explore and validate the practical applications of remote VA testing.

## 1. Introduction

Home monitoring and self-testing are a major current focus in medicine. The COVID-19 pandemic prompted the integration of telemedicine into ophthalmic practice, known as teleophthalmology [1], whereby healthcare providers had to adapt creative approaches to ensure effective diagnosis and treatment while safeguarding patients and medical staff [2]. Remote assessment of visual function is an important area of research that requires the development of home-based, patient-oriented technologies [3].

Remote patient monitoring, facilitated by home-based systems, holds the potential to enhance accessibility to healthcare services and improve patient outcomes across a wide range of ocular conditions, mainly in patients with chronic eye conditions or lack of access to office-based examinations. Additionally, home monitoring can enhance the quality of care, leading to improved outcomes and reduced healthcare costs [4].

Visual acuity (VA) is a fundamental measure of visual function in clinical practice [5]. This is the initial test that is performed in a clinic [6] that gives an indication of refractive error, media abnormalities, macular function, and the integrity of the visual pathways [7]. VA theory revolves around assessing the clarity or sharpness of vision, which is most commonly measured using the Snellen chart. Developed by Herman Snellen in the 1860s, the Snellen chart is a tool featuring rows of black symbols on a white background that decrease in size, used to determine the smallest letters a person can read from a specific distance, typically 20 feet, which represents the minimal angle of resolution. The Snellen chart is commonly used to measure VA in ophthalmology and optometry [7]. Snellen charts may be used at a distance (6 m) or near (40 cm) [8,9]. It is important to recognize that impaired near vision can have a significant impact on quality of life, similar to the effects of decreased distance vision [10]. Furthermore, there is a strong correlation between distance VA and near VA [11], as measured with the Rosenbaum Pocket Vision Screener (RPVS), which is a commonly used Snellen-based near card in ophthalmic practice [3].

Various digital tools have emerged in recent years to monitor patients’ VA [12], including smartphone-based applications such as V@home [13], Peek Acuity [14], GoCheck Kids [1], Sightbook [15], TreC Oculistica [16], the web-based index test [17], the Pocket Vision Screener [18], self-administered tests, such ad as the Early Treatment Diabetic Retinopathy Study (ETDRS) home VA test [2] and the Accustat^®^ [3], and even the first smart TV-based VA test, the Democritus Digital Visual Acuity Test (DDiVAT) [19]. However, though a low mean difference between digital tools and the standard charts and clinical equivalence indications were previously reported, the wide 95% limits of agreement revealed the lower precision of the digital self-assessments, especially in patients with decreased VA [20]. Moreover, in technologically challenged populations, digital tools may not be effective.

The necessity to develop a new VA card test for self-use and home monitoring stems from the limitations observed in both traditional in-clinic assessments and existing digital tools for VA measurement. Traditional methods, such as the Snellen and RPVS methods, require in-person visits to healthcare facilities, posing challenges for individuals with mobility issues, chronic conditions, or those residing in remote areas. Digital tools, despite their innovation, have shown variability in precision, particularly in patients with reduced VA, and may not be accessible or user-friendly for all populations, especially those with technological limitations [20]. Additionally, the implementation of digital VA tools remains underdeveloped, which could significantly improve the applicability of these tests for self-administration or evaluations by non-specialists. [12] The COVID-19 pandemic further underscored the importance of remote healthcare capabilities, including teleophthalmology, emphasizing the need for reliable, non-digital, self-administered VA tests, for populations that are less comfortable with technology or without access to digital devices. The “Hadassah Self-Visual Acuity Screener” (HSVA) was thus developed to address these gaps, offering a practical solution for consistent, accurate home-based VA monitoring and reducing dependency on clinical visits, thereby enhancing healthcare accessibility and efficiency.

The objective of this study was to create and validate a user-friendly non-digital near VA card test, the HSVA, that will enable card-based, self-monitoring of VA. This may be particularly effective for patients living in peripheral areas with limited availability to medical services or patients with chronic or recurring eye diseases, reducing the need for formal, medical-staff-based VA testing during clinic visits and improving patient flow.

## 2. Methods

### 2.1. Sample and Study Design

This cross-sectional study received approval from the institutional Helsinki committee of Hadassah Medical Center (study#: HMO-21-152) and the IRB committee of Hadassah Academic College (study#: HAC-220). Prior to participation, all patients were provided with a clear explanation of this study’s objectives and procedures, and they provided written informed consent. All data were coded and analyzed anonymously.

A cohort of individuals who visited the ophthalmology and optometry clinics, as well as their accompanying individuals, were approached to take part in this study. Patients with ocular or systemic pathologies were included. The exclusion criteria encompassed patients with a best-corrected visual acuity (BCVA) of lower than 1.0 LogMAR (0.1 decimal) in the better-seeing eye, pregnant women, those who refused to participate, and individuals who could not sign the consent form.

### 2.2. Development of the near Visual Acuity Chart

**Figure 1:**
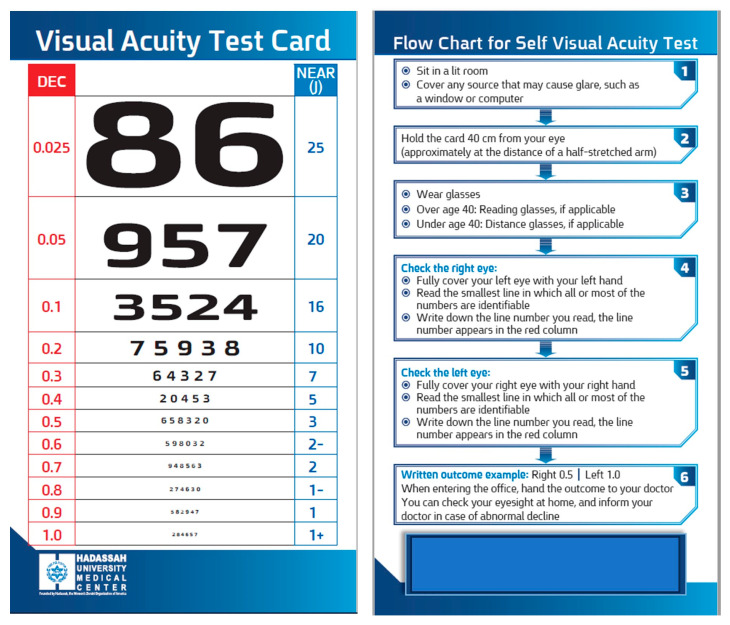
The Hadassah Self-Visual Acuity Screener (HSVA). ©Hadassah Medical Organization, 2022.

A novel card that was termed the “Hadassah Self-Visual Acuity Screener” (HSVA) was developed to facilitate near visual acuity assessment (Figure 1). The dimensions of the optotypes were designed to measure the range of VA that is not covered by the RPVS chart. When it was first developed, the RPVS was designed to be compatible with the Snellen chart proportion of each optotype at each line adjusted to a working distance of 35.56 cm, and the letters on the 20/20 line have a height of 8.73 mm and are viewed at a distance of 6 m [21], yet it does not contain all the current Snellen lines’ acuities (0.3, 0.7, and 0.9 decimal units). In order to address the significant gaps between adjacent lines, particularly noticeable in the smaller-sized numbers, supplementary lines were introduced into the HSVA card. The optotype sizes and line spacings on the HSVA chart were derived from the angular subtense at a standard near reading distance (40 cm), aligning each line with specific visual acuity levels per minimum angular size principles. To address the gaps in existing near VA charts, we averaged the sizes between lines, introducing additional gradations for more accurate near VA assessments, while ensuring consistency with the logarithmic scale of standard VA measurements. Pilot testing with a varied participant group informed adjustments in optotype sizes and spacings, validating the HSVA chart’s effectiveness in near VA measurement based on visual perception standards. Additionally, a column of the corresponding decimal unit VA was incorporated alongside each row (0.3 decimal font size), simplifying the conversion of near visual acuity test results into their distance equivalents. Lastly, the reverse side of the card featured a user-friendly flow chart (0.4 decimal font size) accompanied by written instructions to assist self-testing. Patients with worse VA who could not read the flow chart were instructed to watch the videotaped instructions. 

#### Procedure

During the near test, participants were provided with instructions to wear their habitual reading glasses if they were aged 40 years or older. For participants under the age of 40, if applicable, they were instructed to wear their distance glasses, and each eye was measured separately, with the occlusion of the untested eye. All tests were conducted in standardized conditions (with dim lighting and the computerized Snellen chart calibrated at 6 m, and near tests were performed at 40 cm). Overall, 253 patients were recruited at the ophthalmology clinics of Hadassah Medical Center and an additional 22 at the optometry clinics of Hadassah Academic College. To assess the repeatability of the new VA card, 38 patients underwent testing twice with a one-week interval using the Snellen chart, RPVS, and the HSVA. The BCVA of 237 additional patients was measured by ophthalmic technicians using these three tests. Among these patients, 67 individuals received oral instructions and a guiding video for self-testing with the HSVA card, which was performed independently in the ophthalmologist’s office, and their self-VA results were compared with the examination results conducted by a masked ophthalmic technician, who was unaware of their self-test results and examined them individually. To minimize the possible variability between the five examiners, all tests were conducted by a professional eye clinic team after training the examiners according to a structured protocol. Data on the visual acuity measured in the past months (up to 12 months) by the Snellen method were available for 87 patients from the cohort and were extracted from the electronic medical records. These previous VA measurements were correlated with the results obtained in the current study.

### 2.3. Outcome Measures 

The mean adjusted difference in BCVA (LogMAR units) tested using the HSVA card between the self-administered values and those obtained by a masked ophthalmic technician was used to assess the accuracy of self-testing. Correlation tests were used to compare the VA values obtained by the Snellen, RPVS, HSVA, and previous clinical vision tests. 

### 2.4. Sample Size Calculation

An initial pilot study performed on 49 participants using the distance Snellen chart and the HSVA revealed a sensitivity of 0.85 and a specificity of 0.95 for up to 2 lines of difference between these charts. Assuming a mean difference of 0.021 LogMAR between the office Snellen BCVA and the home self-test of near BCVA [3], a precision of 0.10, a confidence interval of 0.95, and a prevalence of 0.50, a sample size of 109 patients was determined to be sufficient for a statistically significant comparison between the two methods of VA measurement (Arifin WN. Sample size calculator). 

### 2.5. Statistical Analysis

The data normality was tested using the Kolmogorov–Smirnov test, and due to non-normal distributions, non-parametric tests were used. All VA values were converted to LogMAR values for the analysis. Only the right eye of all study participants was included in the analysis [22] due to the high correlation between the VA of both eyes, and if the right eye of a patient had a VA of lower than 1.0 LogMAR, in such a case, the left eye was included. The comparisons of the mean visual acuity results of the Snellen, RPVS, and HSVA methods were analyzed using the Wilcoxon test. Also, the self-test HSVA results, the results obtained by the ophthalmic technician, and the VA values extracted from the medical records were analyzed by the Wilcoxon test. Bonferroni correction was used for post hoc analysis, and for multiple comparisons. Spearman’s and intra-class correlation coefficients (ICCs) were applied. Bland–Altman analysis (95% limits of agreement) was used to assess the agreement between HSVA and RPVS charts. Linear hierarchical regression was applied to predict the Snellen and RPVS acuities obtained by the HSVA test, using univariate analysis as well as multivariate analysis while adjusting for age as a confounder. A statistically significant result was considered as *p* < 0.05 in a two-tailed test. The analysis was performed using SPSS software (Version 27.0, IBM SPSS Statistics, Chicago, IL, USA; IBM Corp, Armonk, NY, USA).

### 2.6. Power Analysis

A study including 38 patients for the repeatability tests yielded a power of 83% with α = 0.05. For agreement calculations between the visual acuity tests, a sample of 240 participants provided a power of 99% with α = 0.05. And 67 patients who were self-tested by the HSVA and were also examined by a masked clinician provided a power of 91% with α = 0.05 (calculated by the G*Power calculator, version 3.1.9.7).

## 3. Results

A total of 275 participants were included, with a mean age of 42.5 ± 19.4 years (range: 18–89 years), and 110 (47%) of them were women. The LogMAR VA ranged between 0.0 and 1.0, with a mean distance VA of 0.15 ± 0.21. 

### 3.1. Test–Retest Reliability

Test–retest reliability analysis was performed on 38 patients (27 women (71%); mean age: 28.2 ± 10.9 years) who were tested twice with a one-week interval on the Snellen, RPVS, and HSVA charts. Similar values were recorded for the two repeated tests of each chart (*p* > 0.05), with very good correlations and repeatability (range: Rs = 0.99 to Rs = 1.0; *p* < 0.001; Table 1).

### 3.2. Accuracy

The comparison between the new HSVA test and conventional clinical (reference) tests revealed the mean distance BCVA (LogMAR) values of an additional 237 participants (140 women (59%); mean age: 42.2 ± 19.4 years) obtained with the Snellen chart (0.15 ± 0.22) were different from those obtained with the RPVS (0.10 ± 0.0; *p* = 0.001) and HSVA (0.09 ± 0.0; *p* = 0.001). Yet, no statistically significant difference was found between the mean RPVS and HSVA results (*p* = 0.10) methods. The ICC of the HSVA with the Snellen chart was 0.33 (Table 2). When age stratification was performed with a median age of 42 years, similar results were obtained: statistically significant differences were found between the Snellen and HSVA methods in participants of <42 years and 42 years and older (*p* < 0.01 and *p* = 0.04, respectively), yet no differences were obtained between the RPVS and HSVA methods in young and old patients (*p* = 0.12 and *p* = 0.32, respectively). 

### 3.3. Agreement between HSVA and RPVS Charts

**Figure 2:**
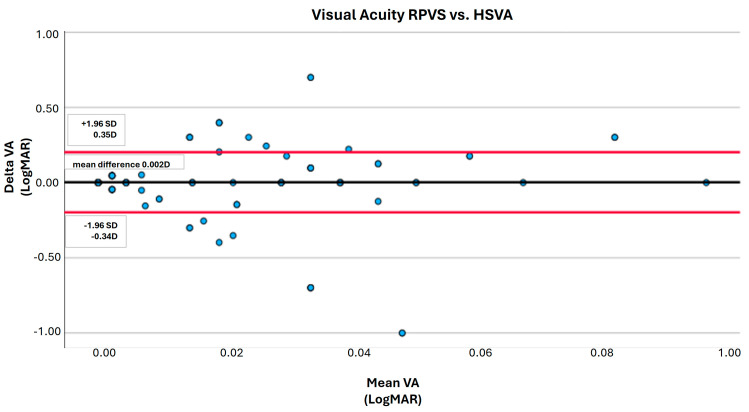
Bland–Altman agreement analysis of the RPVS and HSVA charts for near visual acuity (95% limits of agreement). LogMAR units. RPVS = Rosenbaum Pocket Vision Screener; HSVA = Hadassah Self–Visual Acuity Screener. No significant difference between these charts (*p* = 0.10). Dots represents visual acuity measurements and red lines represents 95% limits of agreement.

Bland–Altman analysis (95% limits of agreement) was performed to assess the agreement between the HSVA and the RPVS cards. When analyzing the linear regression line of the Bland–Altman scatter plot, the HSVA and RPVS values demonstrated no difference of proportional bias in the scatter dots above and below the mean difference line for VA (*p* = 1.0; Figure 2).

### 3.4. Self-Test HSVA

A comparison of the mean HSVA self-test VA results (n = 67 patients; 39 women (58%); mean age: 49.6 ± 20.1 years) with the HSVA results obtained by a masked ophthalmic technician revealed similar values (0.10 ± 0.20 and 0.09 ± 0.19, respectively; *p* = 0.17), a very good agreement (Rs = 0.87; *p* < 0.001), and an ICC of 0.96. (Table 3) Stratification by age revealed no statistical differences between the self-test and the examiner in both age groups (*p* = 0.31).

### 3.5. Self-Test vs. Electronic Medical Records

The distance VA results of 87 patients as documented in their electronic medical records (EMRs) in the year prior to their participation in this study were compared with the HSVA results obtained by an ophthalmic technician, and in 26 of the patients, self-tested VA values were also obtained (Table 4). The results show that similar VA values were recorded using the Snellen distance charts in previous clinic visits and using the near HSVA by a technician (*p* = 0.12). However, a difference was noted between the distance VA extracted from the EMRs and the near self-tests (*p* = 0.04).

### 3.6. Prediction Analysis

Table 5 demonstrates the linear hierarchical regression that was applied to predict the Snellen distance and the RPVS near acuities by the results obtained by the HSVA test. In the univariate analysis, age predicted the near RPVS VA results (β = 0.35; *p* = 0.003; R^2^ = 0.13) as well as the distance Snellen VA results (β = 0.26, *p* = 0.02; R^2^ = 0.07). In the multivariate analysis, while adjusting for age, the model revealed that the HSVA results obtained by a technician predicted the RPVS results with a relatively high accuracy (β = 0.91; *p* = 0.001; R^2^ = 0.88), while the self-test HSVA predicted the distance Snellen VA results (β = 0.93; *p* = 0.01; R^2^ = 0.36). 

## 4. Discussion

Herein, we reported the development and validation of a novel near visual acuity (VA) card test that enables self-monitoring against two gold-standard charts. The main distinctive features of this card are the decimal units that are displayed next to each row, which facilitate the conversion of near VA test results into their distance equivalents; the additional lines compared with the standard cards, especially for the smaller-sized numbers that correspond to better VA; and the flow chart that guides the patients in the self-administration of this test.

The HSVA test showed good test–retest reliability, accuracy, and agreement of the VA assessments with the near RPVS values. These results are consistent with previous studies that reported similar distributions of Jaeger scores and mean LogMAR equivalents between the standard Jaeger test and a new near method (Philippine peso bill) [23]. A recent systematic review of 17 publications that included studies on 13 different digital tools provided a current overview of digital tools for remotely assessing visual function that can be used without assistance of a healthcare professional and evaluated their accuracy. A low mean difference between digital visual acuity assessments and reference charts was reported, suggesting clinical equivalence [20].

In the current study, the HSVA test demonstrated a moderate correlation with the distance Snellen chart results, and it also demonstrated that the self-test in this card could accurately predict the distance Snellen VA results. These findings are supported by a previous study showing a high correlation between the VA measured with the near vision digital self-test and the office Snellen acuity test [3]. Wolffsohn and colleagues reported a high correlation between the distance VA measured with the distance Bailey–Lovie chart and the near VA measured by the near Bailey–Lovie chart, and the Practical Near Acuity Chart (PNAC), which uses a single paragraph with three simple related words on each line, especially when the patient did not have ocular pathology and the test was performed with high contrast [11]. A similar conclusion was reported by another study that showed good repeatability when comparing distance visual acuity with near visual acuity with a small difference (of half a line) [24]. These results indicate that it is feasible to use a near VA test and extrapolate its results to distance VA. 

A key finding of this study was the high correlation between self-test VA by the HSVA and the results obtained by a masked ophthalmic technician. The new HSVA test card was designed to enable patients to self-assess their visual acuity and detect any significant changes that require urgent consultation with an ophthalmologist. Chen and colleagues recently reported an ICC of 0.94, indicating a strong positive correlation between at-home VA measured with the Accustat^®^ near vision digital self-test and the office Snellen test [3]. Similar findings were found even when patients performed self-testing on an ETDRS chart compared with an in-office test [25]. Xian et al. also reported a good agreement between a mobile-application-based VA self-test program and conventional VA tests [26]. Another web-based test was also found to be a valid tool for remotely assessing VA, even when performed independently by patients at home [17]. Even when performing the VA test on a smart TV, there were non-significant VA differences compared with a standard ETDRS test, suggesting that it can be used for accurate VA self-assessment in telemedical settings, both in normal and low-vision patients [19]. A systematic review concerning the utilization of digital instruments for VA assessment revealed a diminished accuracy in digital self-assessments [20]. However, Thirunavukarasu and colleagues conducted an extensive review, concluding that remote self-administered VA tests show potential benefits. They suggest that further pragmatic trials are necessary to substantiate their implementation in specific contexts, which could enhance the utility of these tests for patient-led or non-specialist assessments. Such deployment could significantly contribute to teleophthalmology, facilitate non-specialist eye evaluations, improve pre-consultation triage, and support autonomous, long-term vision monitoring [12].

The overall high agreement between the HSVA self-test results and the results obtained by the masked examiner suggests that the instructions provided on the back of the card in simple language, along with basic guidance from a clinician at the clinic, allows the self-tested patients to obtain valid results. Near VA methods can facilitate self-monitoring of VA even if there is a discrepancy of one or two lines or 4–7 letters between the self-test results and the results measured by the examiner or the distance test [2,3,25]. Though HSVA performance may vary across age groups, particularly among the elderly or those with potential access issues to in-person testing, similar results were obtained when the analysis was performed stratified by age groups. 

Self-administered VA tests like the HSVA enhance patient engagement by allowing frequent at-home monitoring, which is beneficial for early detection of vision changes and timely intervention. These tests streamline the clinical workflow by providing preliminary VA data, aiding in prioritizing and efficiently planning patient care. Contrary to replacing clinician assessments, they supplement them, serving as an additional data point for a more informed and collaborative approach to patient care, thus improving overall outcomes.

Based on the correspondence between near and distance VA, several tools for near VA assessment have been developed for clinical use [27], including apps for self-measurement of VA [28] and a self-test card [29], and their results have been found to be suitable and effective for monitoring changes in VA. Hence, the HSVA, which demonstrated consistent results with good repeatability, and was tested in the current study on patients from tertiary ophthalmology clinics, including patients with various eye pathologies, could serve for self-monitoring to detect changes in VA, particularly in patients with chronic eye diseases that are under recurrent treatment (such as patients receiving repeated intravitreal injections of anti-VEGF medications). The HSVA simplifies the use of the self-VA test with written and videotaped instructions and additional lines in the better-VA-sized numbers, and the decimal units next to each row ascribe this test several advantages that do not exist in other printed VA cards.

One of the limitations of this study is the fact that it was developed as a printed version rather than a digital one. In an era where there are emerging technologies for digital tests and smartphone-based applications to assess visual acuity, it might seem more natural to create another digital tool. However, there are populations that are technologically challenged, such as elderly people with chronic eye conditions, or populations who do not own smartphones due to economic or religious reasons. Thus, to avoid bias and the exclusion of potential populations and to maintain the simplicity of the test, it was developed as a non-digital version. Furthermore, as long as the self-administered at-home test follows a standardized protocol, it is equivalent to a standard technician-administered VA test in a clinic in the examined population [2]. There is a broad agreement that both smartphone apps and printable materials assessing VA are easy, intuitive to use for patients, and reliable for clinicians [16]. Investigating the feasibility of remote testing and its value in enhancing patient convenience and healthcare accessibility, future research is setting up a framework for the remote application of the HSVA test. Moreover, a direct comparison of the HSVA with digital devices would provide valuable context and evidence of the HSVA’s utility. 

Additionally, although the Early Treatment Diabetic Retinopathy Study (ETDRS) chart is regarded as a more reliable measure, the present study employed the Snellen chart for comparative purposes. This approach is customary in the validation of novel VA charts, where the Snellen chart is utilized as a reference [12]. Furthermore, despite the absence of a standardized protocol for employing Jaeger notation in contemporary near reading cards [30], this metric was adopted due to its continued clinical acceptance and widespread use in numerous countries for daily visual assessment [23].

Another limitation of the HSVA chart is its moderate correlation with standard distance VA tests. Ideally, a high correlation would suggest that these tests could serve interchangeably, offering a clinically practical, patient-operable alternative to traditional distance VA assessments. However, the HSVA is designed to supplement, rather than supplant, existing distance VA evaluations. Notably, the HSVA demonstrates a very strong agreement with the RPVS, underscoring its efficacy in near VA screening. This strong concordance highlights the HSVA’s capacity to detect significant VA changes in home monitoring, thereby prompting patients to seek a comprehensive examination when necessary.

However, this study aimed to demonstrate a good agreement between the HSVA and the widely accepted near test with the RPVS, as well as a close similarity between the self-test results and the results obtained at the clinic. Accuracy was indeed identified in these two parameters, suggesting that substantial VA changes (>two ETDRS lines) would be identified by both the near and the far test [18]. Finally, self-administered VA tests may extend their utility beyond the field of ophthalmology, finding relevance in primary care, emergency medicine, and neurology. Further investigation is warranted to delineate the feasible applications of remote VA assessments. While in-person evaluations remain paramount for exhaustive ophthalmological examinations, remote VA testing could enhance healthcare delivery and alleviate pressures on constrained clinical resources, particularly when integrated with other nascent digital health technologies. Validated self-administered VA tests hold promise in enhancing teleophthalmology services, facilitating pre-consultation triage, enabling long-term visual monitoring, and supporting the assessment and documentation of ocular conditions by non-specialists.

## 5. Conclusions

In this study, a new card for the self-test monitoring of near VA was developed and validated. The HSVA incorporates decimal units, which are absent in standard near cards, as well as additional lines to assess high levels of VA, simple instructions, and a video guide for self-testing. Moreover, this study evaluated the agreement of the card with an existing near test and the patient’s self-testing ability, and, therefore, it can be applied in clinical settings, but with caution, considering that it does not fully represent distance VA. In addition, this study was conducted on participants in a tertiary clinic who represented a wide range of patients with visual impairments, including patients with various eye pathologies, so it may suggest generalizability to healthy subjects in the community. This test may be useful for many patients with chronic eye conditions or limited access to office-based examinations. 

## Figures and Tables

**Table 1:** Repeatability analysis of two repeated tests on the Snellen, RPVS, and HSVA visual acuity charts (N = 38).

Visual Acuity Chart	Mean	Median	SD	*p* *	Spearman (Rs)	*p*
Snellen—1st test	0.06	0.0	0.19	0.14	0.99	**<0.001**
Snellen—2nd test	0.04	0.0	0.14			
RPVS—1st test	0.02	0.0	0.11	1.0	1.0	**<0.001**
RPVS—2nd test	0.02	0.0	0.11			
HSVA—1st test	0.02	0.0	0.11	1.0	1.0	**<0.001**
HSVA—2nd test	0.02	0.0	0.11			

* Wilcoxon test. LogMAR units. RPVS = Rosenbaum Pocket Vision Screener; HSVA = Hadassah Self-Visual Acuity Screener. Bold represents statically significant result.

**Table 2:** Comparison of the VA test results by the Snellen, RPVS, and HSVA charts (N = 237).

Visual Acuity Chart	Mean	Median	SD	*p* *
Snellen	0.15	0.1	0.22	Snellen–RPVS: **0.001**
RPVS	0.10	0.0	0.19	Snellen–HSVA: **0.001**
HSVA	0.09	0.0	0.20	RPVS–HSVA: 0.10

* Wilcoxon test. LogMAR units. RPVS= Rosenbaum Pocket Vision Screener; HSVA= Hadassah Self-Visual Acuity Screener. Bold represents statistically significant result.

**Table 3:** Comparison of the visual acuity obtained by self-testing and by an ophthalmic technician using the HSVA card (N = 67).

Test Method	Mean	Median	SD	*p* *	Spearman (Rs)	*p*	ICC
Self-test	0.10	0.0	0.20	0.17			
Masked examiner	0.09	0.0	0.19		0.87	**<0.001**	0.96

* Wilcoxon test. LogMAR units. HSVA = Hadassah Self-Visual Acuity Screener; ICC = Inter-Class Correlation. Bold represents statistically significant result.

**Table 4:** Comparison of the near HSVA and the distance Snellen visual acuity extracted from electronic medical records (N = 87).

Visual Acuity Chart	Mean	Median	SD	*p* *	*p* *
Snellen (medical records)	0.24	0.1	0.31	0.12	
HSVA by examiner	0.18	0.0	0.27		**0.04**
Self-test HSVA (N = 26)	0.19	0.05	0.27		

* Tested by Wilcoxon test. LogMAR units. HSVA = Hadassah Self-Visual Acuity Screener. Bold represents statistically significant result.

**Table 5:** Linear hierarchical regression for visual acuity of RPVS and Snellen charts using the HSVA card by self-test and by examiner (N = 67).

		Near RPVS VA			Distance Snellen VA		
		B (SEB)	β	*p*	B(SEB)	β	*p*
**Model I**	Age	0.004 (0.001)	**0.35**	**0.003**	0.011 (0.07)	**0.26**	**0.02**
	F	9.5		0.003	5.1		0.02
	R^2^	0.13			0.07		
**Model II**	Age	0.001 (0.00)	0.08	0.06	0.001 (0.001)	0.09	0.37
	HSVA by examiner	0.13 (0.18)	**0.91**	**0.001**	−0.50 (0.45)	−0.40	0.27
	HSVA self-test	−0.24 (0.17)	−0.22	0.15	1.10 (0.43)	**0.93**	**0.01**
	F	156.9		0.001	11.6		0.001
	R^2^	0.88			0.36		

VA = visual acuity; RPVS = Rosenbaum Pocket Vision Screener; HSVA = Hadassah Self-Visual Acuity Screener. Bold represents statistically significant result.

## Data Availability

The datasets generated and/or analyzed during the current study are available from the corresponding author upon reasonable request. Additional details regarding the research materials, including the development and instructions for the Hadassah Self-Visual Acuity Screener (HSVA), can also be provided upon request. All data are stored in accordance with ethical guidelines and privacy regulations to ensure the confidentiality and integrity of participant information.

## Abbreviations

VA	Visual acuity
RPVS	Rosenbaum Pocket Vision Screener
HSVA	Hadassah Self-Visual Acuity Screener
BCVA	Best-corrected visual acuity
ICC	Intra-class correlation coefficient
ETDRS	Early Treatment Diabetic Retinopathy Study
DDiVAT	Democritus Digital Visual Acuity Test
PNAC	Practical Near Acuity Chart
